# Treatment for Fistula in Ano without a Cutting Operation

**Published:** 1893-04

**Authors:** T. J. Bennett

**Affiliations:** Austin, Texas


For Daniel's Texas Medical Journal.

### A TREATMENT FOR FISTULA IN ANO WITHOUT A CUTTING OPERATION.

BY T. J. BENNETT, M. D., AUSTIN, TEXAS.

(Read before the Austin District Medical Society, March 23, 1893.)

AT THE San Antonio meeting of the Texas State Medical Association, held in April, 1889, I presented to that body a short paper with the above title, in which I endeavored to explain a very simple procedure for the relief of fistula in ano, basing the claim for the treatment upon the report of five or six well selected, successful cases. This paper was very short, scarcely occupying two pages of the Transactions. Either from its being buried in this volume, or for want of real merit in the suggestion (the latter most probably), the little idea never set the world cn fire; it "died a bornin'," for I have kept my eyes on the journals, expecting a thrill, for four solid years, and "nary a thrill." But during these years I have had more experience with the method, and I am convinced of its value as a procedure for the cure of this very annoying and painful trouble.

The operation consists in first thoroughly cleansing the lower bowel and the fistulous tracts with an antiseptic wash, by means of a suitable syringe. I use for the sinuses a long nozzled uterine syringe, capable of considerable force, and a twenty-five per cent. solution of peroxide of hydrogen, or a simple carbolized solution, with which the tracts are freely flooded. The next step is to thoroughly divulse the sphincter muscle, in the usual way, with the fingers. Then again irrigate the sinuses in the same careful manner as before, with the same solution, and last, with plain water. After this, inject into the tracts a solution of nitrate of silver, forty to sixty grains to the ounce of water. Then with a little carbolized oil, or vaseline, spread upon absorbent cotton and bound to the parts with a bandage, the operation is completed, in simple cases.

In cases of the internal blind variety of fistula, openings should be made from the outside with the knife, in order that the pouches which are always formed at the termini of such tracts, and which always contain pus and debris, may be washed out, and the sinuses cleansed. The only cutting necessary is in this variety, and possibly the enlargement of some of the external openings for the nozzle of the syringe, which is introduced only far enough to inject the fluid. With an experience so limited as mine has been, it would be presumptuous in me to claim to have discovered a treatment that would supercede the long tried and successful methods of our text-books. I can only say that the above plan is simple, and so far as I have tried it, it has been successful in every instance. Even a case of recto-vaginal fistula operated on in the same manner, recovered in a few days. In this case, I may say that I employed a six or seven per cent. solution of carbolic acid, instead of the silver, because I did not have the latter solution with me, not knowing or thinking that I should be called upon to do more than lance an abscess, as I had done before for this patient on two occasions, within a year.

The time saved to the patient in this operation, constitutes one of its chief advantages. The number of days required to remain in the recumbent position is from four to eight, and with no wounds to dress and keep clean, the patient spends this time in comparative comfort.

I wish it understood that I do not claim anything original in this operation, except, possibly, that I have placed together in an unusual manner some well known methods and principles.

The necessity of thoroughly cleansing the sinuses, getting rid of all foreign particles and matter, has been recognized and appreciated from almost the beginning of time. Also the divulsion of the sphincter has been employed, and some good results have been reported from this alone. And, too, the injecting of irritants into fistulous tracts has been resorted to, and reports have been made of cures resulting from this alone.

But the arrangement of these things in the manner and order given, belongs to me, so far as I know. The rationale of this treatment is based upon some well established surgical principles, together with a reasonable appreciation of the anatomy, pathology and physiology of the parts involved. In the first place, we have to deal with the most dependent portion of a long and tortuous tube, the blood supply of which is influenced by gravitation, and whose return flow is through veins that are without valves, and against gravity; with a mucous membrane that is in a constant state of hyperæmia, existing in all persons, it is claimed, whether they have ever had a rectal disease of not. And further, we have a large, ring-shaped muscle, the sphincter, which has scarcely any bony attachments, and whose fibers are closely adherent to the integument, and blended more or less intimately with the other muscles and structures about the anus. This muscle stands vigilant guard over the great outlet of the human body. It is quite easy, then, to understand that the sphincter is very important, and that it is liable to become wonderfully hypertrophied from inflammatory stimulus during disease of these parts. The longer the disease continues, whether hemorrhoids, fissures, or fistulæ, the greater the amount of blood to the parts, and consequently the larger the muscle grows and the stronger it becomes. Thus with increased size and strength, there is a corresponding retardation of the return circulation which favors disease, and above all, there is a wonderful increase in the muscular tension of the whole ano-gluteal region. The voluntary power of this muscle is diminished, and the involuntary power is proportionately increased, which also favors disease. Now, a fistulous tract, it matters not what produces it,—a foreign body that has lodged above the sphincter, a traumatism, constipation, or what not, anything, in fact, that would cause an abscess—for an abscess under all circumstances, it is claimed, causes the sinus,—this fistula, except in the superficial form, has its origin above the sphincter, within the bowel, and extends, more or less tortuously, down by the side of, and exterior, to the muscle, and makes its exit through the skin within an inch or two of the anus. Let there be many of them, or only a few, the destructive process is the same in all. The high tension of the muscular tissue causes retraction of the severed fibers, in accordance with the only function of muscular tissue, viz: contraction, and the result is non-coaptated walls. It may be likened to a taut suspender. As the rubber threads are cut, they retract, leaving an eliptical, or round-shaped hole. So in the case of the fistula in ano. Fecal matter, when in a liquid state, is always present in these sinuses, and prevents, together with the abnormal tension of the parts, a union by any plan of treatment other than cleansing, and relaxing the tension. This removes the debris, and permits the coaptation of the walls. The nitrate of silver is used simply to hasten union, which it does by exciting a temporary inflammation along the tract, thereby causing a cure before the paralyzed sphincter can regain itself. This is exactly what is done when all of the holes are cut into one, the standard operation for this trouble. The sphincter is severed, which relaxes the surrounding parts, and permits the cleansing of the tracts and the coaptation of the walls. Now, it is not proposed to do more than to state what has been the results of my limited experience with the operation which I have had the honor to present to you to-day, and I may be permitted to summarize the advantages claimed as follows:

1. There is from one week to a month saved in time to the patient.

2. There is less danger from incontinence.

3. There is no danger from embolism.

4 There is no suppurating wound to require daily dressing and cleansing.

5. The operation is practically robbed of its terror.

Certainly, then, the procedure is simple, and, to my mind, reasonable, and therefore I respectfully commend it to your further investigation and trial.

